# Role of Solid Wall Properties in the Interface Slip of Liquid in Nanochannels

**DOI:** 10.3390/mi9120663

**Published:** 2018-12-16

**Authors:** Wei Gao, Xuan Zhang, Xiaotian Han, Chaoqun Shen

**Affiliations:** 1 Key Laboratory of Energy Thermal Conversion and Control of Ministry of Education, School of Energy and Environment, Southeast University, Nanjing 210096, China; weigao@seu.edu.cn (W.G.); cathyxuan@seu.edu.cn (X.Z.); 2 School of Hydraulic, Energy and Power Engineering, Yangzhou University, Yangzhou 225127, China; xthan@microflows.net

**Keywords:** slip, interface, surface, rough, nanochannel

## Abstract

A two-dimensional molecular dynamics model of the liquid flow inside rough nanochannels is developed to investigate the effect of a solid wall on the interface slip of liquid in nanochannels with a surface roughness constructed by rectangular protrusions. The liquid structure, velocity profile, and confined scale on the boundary slip in a rough nanochannel are investigated, and the comparison of those with a smooth nanochannel are presented. The influence of solid wall properties, including the solid wall density, wall-fluid coupling strength, roughness height and spacing, on the interfacial velocity slip are all analyzed and discussed. It is indicated that the rough surface induces a smaller magnitude of the density oscillations and extra energy losses compared with the smooth solid surface, which reduce the interfacial slip of liquid in a nanochannel. In addition, once the roughness spacing is very small, the near-surface liquid flow dominates the momentum transfer at the interface between liquid and solid wall, causing the role of both the corrugation of wall potential and wall-fluid coupling strength to be less obvious. In particular, the slip length increases with increasing confined scales and shows no dependence on the confined scale once the confined scale reaches a critical value. The critical confined scale for the rough channel is larger than that of the smooth scale.

## 1. Introduction

The microscale heat and mass transfer has recently been an attractive subject of both scientific investigations [1] and engineering applications, such as microfluidic preparation [2,3,4], biomedical detection [5,6], surface engineering [7], microelectronic cooling [8,9], microchemical production [10,11,12], micro-energy technology [13,14], micro-heat transfer devices [15,16], etc. When the dimensions of the microfluidics system reach the nanoscale, a “no-slip” boundary condition is assumed. This assumption, at the walls of a flowing liquid, has been challenged in the modeling of the liquid flow [17,18,19]. At this scale, owing to the strong liquid-solid interaction inside a confined nanospace, the interface slip of liquid at the boundary is crucially affected by the solid wall properties of the channel [20]. The roughness is a basic feature of a solid wall, since the surface profile is impossible to be smooth in actuality. In addition, the fabrication of nanochannels with appropriate aspect ratios with the incorporation of mechanical, chemical, or electrical properties poses a unique challenge [21,22]. The interfacial slip behavior is a key mechanism for addressing this challenge. Therefore, it is significant to understand the role of the solid wall on the interface slip of liquid in rough nanochannels.

Since the 1950s, there has been a series of experimental studoes on the interface slippage of liquid on a solid surface. For example, Watanabe [23] observed an increase of mercury flow in a glass capillary (radius 3.5 and 13.3 microns) arising from the effect of a boundary velocity slip. Churaev et al. [24] measured the flow of water and mercury in a quartz capillary and showed that the degree of wall slip gradually decreases as the temperature increases. With the recent rapid development of microscale measurement technology, the experimental work of liquid slip on a solid surface has become studied more in-depth. Surface force apparatus [25], atomic force microscopy [26], particle image velocimetry (PIV) [27], and other advanced technologies were applied to measure the liquid flow slip behaviors on a solid surface. For example, Pit et al. [28] used fluorescence reflection recovery camera technology to visually observe the velocity slip of liquid on a solid surface with hexadecane as a working fluid. Bhushan et al. [26] used atomic force microscopy to measure the slip length of hydrophilic and hydrophobic surfaces, in which the values of slip length were 43 nm and 236 nm, respectively. This implies that the hydrophobic surface will be more conducive to the slippage of the liquid on the solid surface. Although experiments have confirmed the existence of the velocity slip phenomenon on a solid surface, the mechanism of velocity slip at a liquid-solid interface still must be further revealed to achieve the potential application of flow drag reduction.

With the recent rapid development of computational science and technology, microscopic simulation has become an effective tool for understanding the microscopic behavior of liquid-solid interaction, especially the interface velocity of liquid on a solid wall [29,30,31]. It is documented that the nanoscale flow behaviors are mainly dependent on surface roughness [32,33,34] and wettability [35,36], liquid properties [37], and shear rates [34,38,39]. Thompson et al. [38] numerically investigated the liquid flow in a Couette flow system by the molecular dynamic method and identified three kinds of boundary conditions, including no-slip, pure slip, and multi-layer locking. Zhang et al [40] conducted a systematic investigation on the effect of roughness elements on the laminar flow in microchannels. It was observed that the flow reversed near the roughness elements, and the vortex-like structures formed in the channels as the flow proceeded over the roughness elements. Wan et al. [41] studied the slip length of liquid flow through rough solid-liquid interfaces in a restrained space using perturbation expansion and the Dyadic Green function, and the results showed that the total slip length at a solid-liquid interface is proportional to the slip length arising from the chemical interaction. Shu et al. [31] explored the slip mechanism of fluid on a solid surface in terms of surface diffusion and proposed a re-adsorption mechanism for interface velocity slip. The previous investigations confirmed that the solid wall properties highly affected the liquid flow behaviors at the interface. Actually, the solid surface on the atomic scales is almost impossible to make smooth, representing that surface roughness is an important factor to affect liquid flow through the solid wall. Sofos et al. systematically investigated the slip flow in nanochannels confined by face center cubic (FCC) arranged, spring force constrained solid rough walls. They provided detailed results to show the effects of roughness geometry, wall wettability, and fluid density [42,43]. Rahmatipour investigated the argon Couette flow through oscillatory walls with rectangular and triangular roughness by molecular dynamics and found that the increase of roughness height would cause reduction of flow slip [44]. Jing and Bhushan [45] reviewed the coupling effect of surface charge and velocity slip in nanochannels and derived a theoretical model to combine the electric double layer, slip, and fluid drag. Prakash et al. numerically simulated the KCl electrolyte solution flow in a nanochannel confined by charged amorphous silicon with random roughness, while the wall-fluid interaction and surface wettability of such walls were measured by Atomic Force Microscopy (AFM) techniques [46,47]. To clarify the function of surface roughness in nanoscale liquid flow, literature tried to analyze an interface velocity slip with surface roughness as characterized by a triangular, rectangular, and sinusoidal distributions, as well as a fractal profile [48,49,50]. The surface topography is significant in the momentum transfer near the wall. However, the effect of solid wall properties on the interface velocity slip for the liquid flow in rough nanochannels still needs to be explored.

Therefore, a molecular dynamics model based on liquid flow past a rough nanochannel is established to investigate the role of a solid wall on the interface slip of liquid in nanochannels with surface roughness constructed by rectangular protrusions. Based on a Poiseuille flow system, the influence of confined scale, solid wall density, wall-fluid coupling strength, roughness height and spacing on the interface velocity slip, and liquid flow, behaviors in rough nanochannels are examined and analyzed.

## 2. Mathematical Model

**Figure 1:**
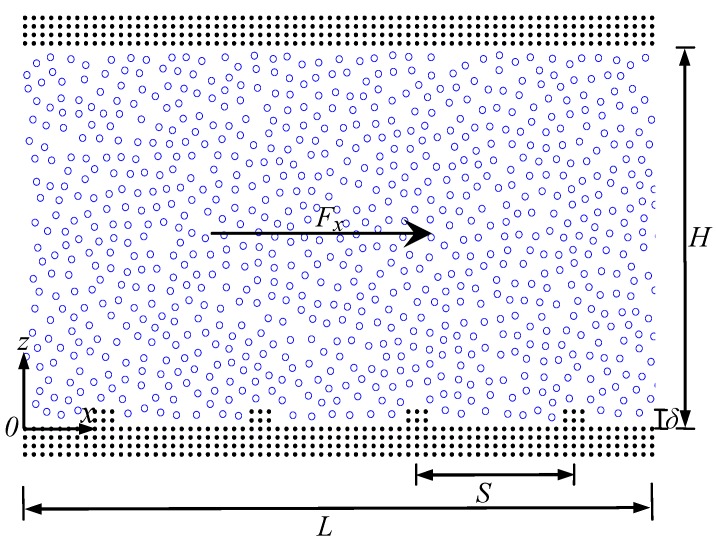
Schematic of Poiseulle flow in the rough nanochannel.

To understand the effect of solid wall properties on the velocity slip at the liquid-solid interface inside a nanochannel, two-dimensional molecular dynamic simulations are performed to simulate Poiseuille flow in rough nanochannels. For liquid flow within the nanochannel, the bottom solid wall is rough, while the top solid wall is smooth. In the current study, the wall roughness is patterned by rectangular protrusions through the addition of extra solid atoms. As illustrated in Figure 1, *δ* denotes the height of surface roughness, *S* denotes the roughness spacing, *H* denotes the confined scale, and *L* denotes the channel length. The wall atoms are clamped at a fixed lattice position by a harmonic spring which possesses a large spring constant. Inside the nanochannel, the interaction of two liquid atoms with a distance *r* can be described by the Lennard-Jones (*L*-*J*) potential [51].

(1) $$\varphi_{LJ}(r)=4\varepsilon \left[{\left(\frac{\sigma }{r}\right)}^{12}-{\left(\frac{\sigma }{r}\right)}^{6}\right]$$

where *ε* is the depth of the potential well for liquid phase and *σ* is the length scale of the liquid phase. In the simulation, the reciprocity between wall atoms and liquid atoms is also described by the *L*-*J* potential with *ε_wl_* and *σ_wl_* to characterize the energy and length scale. To save the computation time, a cutoff radius, *r_c_* = 2.5*σ*, is employed for the *L*-*J* potential. The liquid system at constant temperature inside the nanochannel is accomplished using a Langevin thermostat. The motion equation of the *i*th liquid molecule is

(2) $$m\ddot{z}=\sum_{j\neq i}\frac{\varphi_{LJ}}{\partial z_{i}}-m\Gamma \dot{z}+\eta_{i}$$

where Γ is a friction constant which governs the rate of heat exchange with the reservoir and *η_i_* is a Gaussian distributed random force. The motion equation is integrated by the use of the Verlet algorithm [51], in which a time step Δ*t* = 0.001*τ* (*τ* denotes the characteristic time of *L*-*J* potential, *τ =* (*mσ*^3^/*ε*)^1/2^) is adopted to simulate the liquid motion. Note that recent molecular dynamics work has seen significant advancement with methods and energy potentials progressing much beyond the standard *L*-*J* potentials used in this paper. The use of more realistic materials and the incorporation of more advanced model approaches can be seen in work by Narayan Aluru [52,53] at University of Illinois and in work by Harvey Zambrano [54,55] at University of Concepcion in Chile.

In the molecular dynamic simulation, the temperature of liquids inside the nanochannel is held at *T* = 1.1 *ε*/*k_B_* (*k_B_* denotes the Boltzmann constant), and the number density of the liquid phase is *ρσ*^3^ = 0.81. A constant external driving force, *F_x_* = 0.02 *ε*/*σ*, along the *x* direction is applied to the liquid atoms to drive the liquid flow through the nanochannel. To explore the effect of solid wall properties on the interface slip, the interfacial parameters are varied by changing the wall-liquid coupling strength, *ε_wl_*/*ε*, and the wall and liquid density commensurability, *ρ_w_/ρ.* All the simulation parameters of the wall geometry, roughness, and fluid-solid interaction are listed in Table 1.

## 3. Results and Discussion

**Figure 2:**
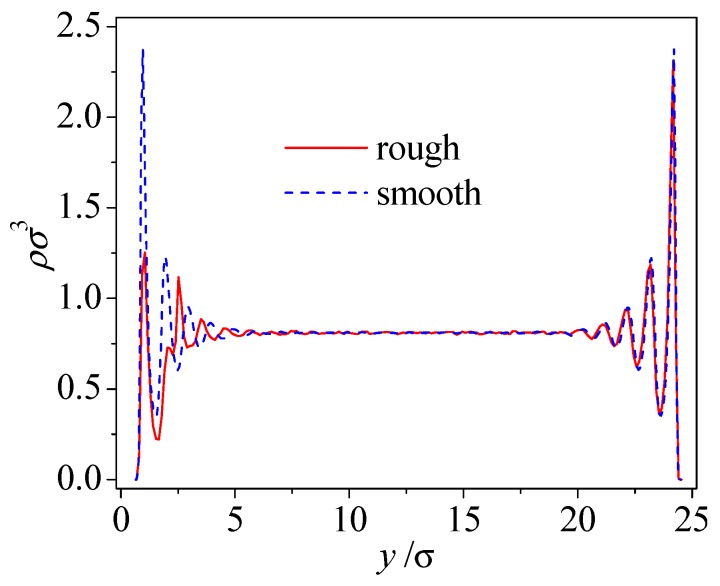
Density distributions in the smooth and rough nanochannels (*ρ_w_* = 2*ρ*, *ε_wl_* = *ε*, *S* = 9.4σ, *δ* = 1.57σ).

The presence of wall roughness affects the liquid microscopic structure in terms of the liquid layers near the wall. Figure 2 compares the normal density profile to the solid wall in smooth and rough nanochannels. As shown in the figure, although there is no difference in density oscillations near the smooth upper solid wall, the rough lower surface induces a smaller magnitude of the density oscillations when compared to the smooth solid surface. The reduced layering reflects the intrusion of the wall roughness into the liquid phase, thus resulting in irregular liquid flow close to the solid surface, which plays a significant role in momentum transfer between the liquid molecules and solid molecules.

**Figure 3:**
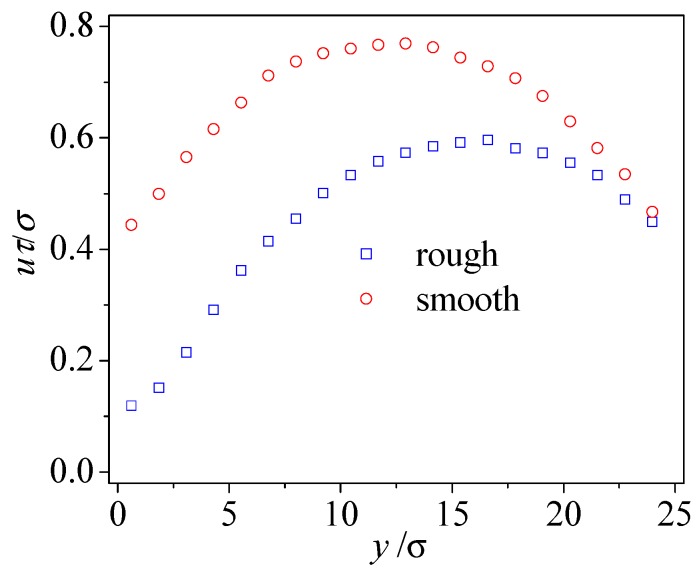
Velocity distributions in the smooth and rough nanochannels (*ρ_w_* = 2*ρ*, *ε_wl_* = *ε*, *S* = 9.4σ, *δ* = 1.57σ).

To further understand the effects of surface roughness on nanochannel flow, Figure 3 compares streaming velocity profiles between the rough and smooth nanochannels. When compared to the smooth nanochannel, liquid flow through the rough nanochannel produces extra energy losses. This is owed to the rough wall, and thus leads to the reduction in both the slip velocity at the rough boundary and the existing velocity level inside rough nanochannel. Despite a similar finite slip velocity at the upper smooth boundary, the velocity profile inside the nanochannel is highly influenced by the presence of the rough wall. The velocity profile is no longer asymmetrical, and is parabolically fit in rough nanochannel.

**Figure 4:**
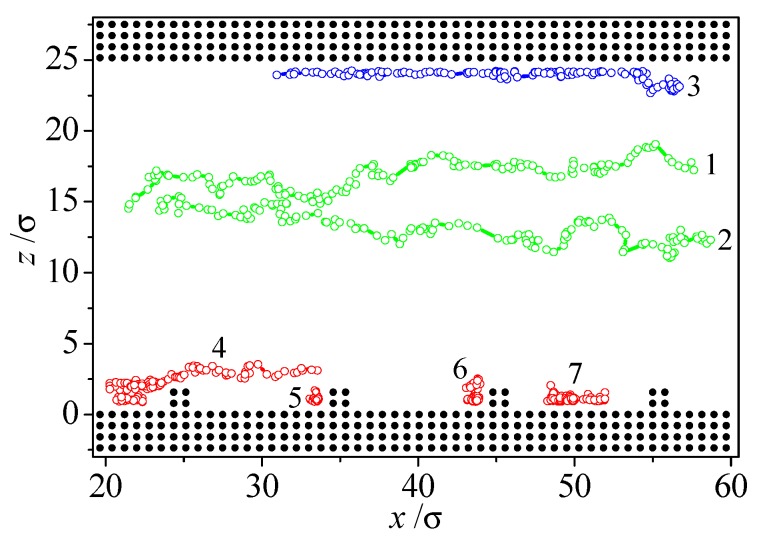
Typical liquid atom trajectories in a rough nanochannel (*ρ_w_* = 2*ρ*, *ε_wl_* = *ε*).

To give a clearer understanding of the wall roughness effect on nanoscale liquid flow, the typical atom trajectories are illustrated in Figure 4. Due to the solid-fluid interaction, the velocity of liquid atoms close to the solid wall is far faster than those in the center region of the nanochannel. In addition, the roughness further confines the molecular motion of fluid near the solid wall and results in a smaller motion space. As shown in the figure, the molecules in the neighborhood of smooth surface make a slower motion, while the atom in the valley of the rough wall only circulates inside the valley and is unable to permeate the interior region of nanochannel during this period. By tracing the flow trajectory of fluid atoms, it is observed that the average trapping time of fluid atoms within protrusions is larger compared with the smooth surface. This result is consistent with a similar case simulated by Sofos et al [42].

To evaluate how the solid wall properties affect the interface slip for the nanoscale liquid flow, it is required to characterize the velocity slip degree for the liquids at the solid boundary. In this paper, the slip length, defined by the linear Navier model, is introduced to describe the interface velocity slip inside a nanochannel.

(3) $$l_{s}=u_{w}/(\frac{du_{x}(z)}{dz}){|}_{w}$$

where *u_w_* is the velocity of the liquid phase on the solid wall, and the subscript *w* indicates the wall. According to Equation (3), the usual no-slip boundary occurs for liquid flow inside the nanochannel when *l_s_* = 0, the presence of interface slip for liquids at solid wall for *l_s_* > 0; *l_s_* < 0, represents that the liquid is locked in the solid wall.

**Figure 5:**
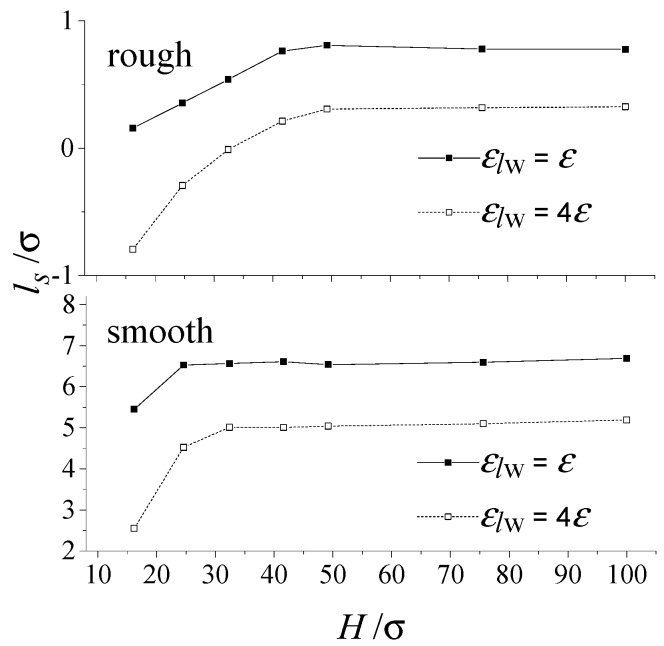
Effect of the confined scale on the interface slip (*ρ_w_* = 2*ρ*, rough: *S* = 19.6σ, *δ* = 1.57σ).

Figure 5 compares the effect of a confined scale on interface slip between the rough and smooth nanochannels. As shown in the figure, regardless of whether the nanochannel is smooth or rough, the slip length increases with increasing confined scales. The slip length shows no dependence on the confined scale once the confined scale reaches a critical value. Under each constant confined scale, the slip length of the smooth nanochannel is far larger than that of the rough nanochannel. It should be noted that the critical confined scale for the rough channel is larger than that of the smooth scale. For the liquids inside the confined nanospace, a larger portion of liquid molecules is on the liquid-wall interaction when the confined scale decreases, indicating that the interface slip behavior of liquid is more easily affected by the solid wall for a smaller nanochannel. However, the enhancement of slip behavior by expanding the size is in effect only within a certain channel length scale.

**Figure 6:**
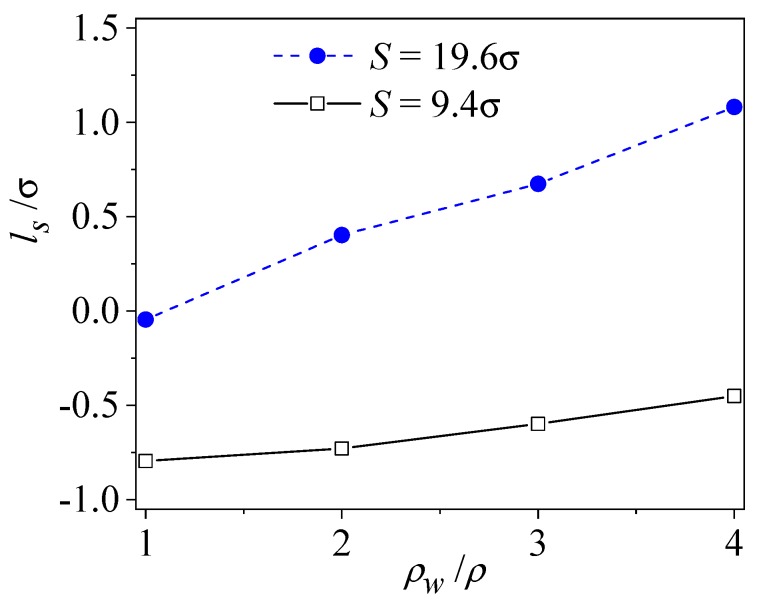
The effect of wall density on slip length (*ε_wl_* = *ε*, *δ* = 1.57σ).

The wall density is one of most important solid wall properties. Compared to the equal wall and fluid densities, the liquid molecule is most preferentially attracted to the solid wall. Conversely, if the wall density is far greater than the fluid density, no fluid molecule could fit in the compact wall. Figure 6 presents the effect of wall density on the interface slip in the rough channel. The increases in wall density lead to a weak corrugation at the atomic level in the surface potential such that the slip length increases, and the maximum slip occurs at the wall with an infinite density. It is also indicated by the figure that, when the roughness spacing is small, irregularities of near-surface liquid flow induce large viscous dissipation, which suppresses the effect of the corrugation of wall potential.

**Figure 7:**
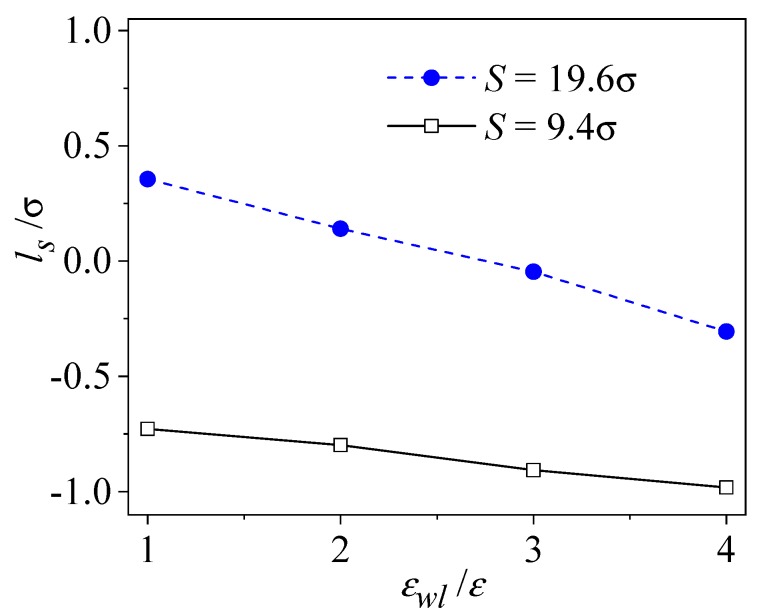
The effect of wall-fluid coupling strength on slip length (*ρ_w_* = 2*ρ*, *δ* = 1.57σ).

The wall-fluid coupling strength is the other most important solid wall property which directly exhibits the solid-fluid interaction. Figure 7 shows the effect of the wall-fluid coupling strength on the interface slip. As seen from the figure, the slip length of the liquid flow at the liquid-solid interface decreases monotonically with *ε_wl_*/*ε*, since a stronger wall-liquid coupling allows the liquid particles closer to the solid wall, even allowng intrusion into the solid wall. Additionally, the interface slip is less sensitive to the wall-fluid coupling strength for the rough wall with small roughness spacing, since irregularities of the near-surface liquid flow dominate the momentum transfer amount at the liquid-solid interface.

**Figure 8:**
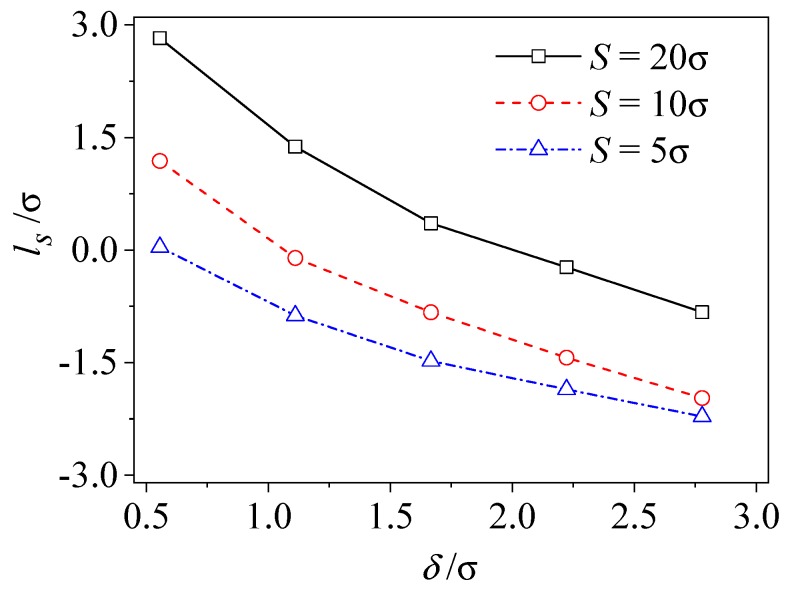
The effect of roughness height and spacing on slip length (*ρ_w_* = 4*ρ*, *δ* = 0.25σ).

In addition to wall density and wall-fluid coupling strength, the roughness height and spacing, which directly represents the solid wall topography, are the two most important solid wall properties. Figure 8 presents the influence of roughness height *δ* on slip length with different spacing *S*. It can be found that the slip length of liquid at the liquid-solid interface is affected by both the roughness height and the roughness spacing. As the statistical roughness height increases, there is a monotonous decreasing trend for the slip length. The slip length can be negative at some points. A higher roughness height causes a larger energy loss for liquid particles outside of the wall cavity. In addition, a rough surface with small spacing introduces additional viscous dissipation, which causes the reduction of the slip degree at the interface. In summary, within the range of present simulation parameters as shown in Table 1, the slip length, *l_s_*, takes a value in the range of −2*σ*~3*σ*. The range of *l_s_* agrees well with the range −4*σ*~4*σ*, given by Galea et al [56].

## 4. Conclusions

The molecular dynamics method is utilized herein to simulate the liquid flow in rough nanochannels in an effort to understand the interface velocity slip. In the simulation, the wall roughness is constructed by rectangular protrusions through the addition of extra solid atoms. Both the liquid-liquid and solid-liquid interactions for the nanochannel flow are described by the Lennard-Jones potential. The liquid structure, velocity profile, and confined scale on boundary slip are presented and compared to those of the corresponding smooth channel. In addition, the solid wall properties, including the solid wall density, wall-fluid coupling strength, roughness height, and spacing on interface velocity slip are all examined and discussed. The major conclusions are drawn as follows:

1. As compared to the smooth atomic surface, the rough surface induces a smaller magnitude of the density oscillations, and liquid flow on the rough solid wall induces extra energy losses, which reduce both the interface velocity of liquid at the rough boundary and the whole velocity level in rough channel.
2. Regardless of whether the nanochannel is smooth or rough, the slip length increases with increasing confined scales and shows no dependence on the confined scale once the confined scale reaches a critical value. The critical confined scale for the rough channel is larger than that of the smooth scale.
3. The decreases in the wall density lead to a strong corrugation at the atomic level in the surface potential, and hence contributes to a reduction of the boundary slip. The slip length also decreases with the wall-fluid coupling strength, since a stronger wall-liquid coupling allows the liquid particles closer to the solid wall. For a smaller roughness spacing, the near-surface liquid flow is more irregular, which induces large viscous dissipation in rough nanochannels, causing the effect of both the corrugation of wall potential and the wall-fluid coupling strength to be less obvious.
4. The slip length for liquid at the liquid-solid interface is influenced by both roughness height and roughness spacing. As the roughness height increases or the roughness spacing decreases, there is a monotonous decrease trend for the slip length, and the slip length can be negative at some points.

## Figures and Tables

**Table 1:** Simulation parameters of the wall geometry, roughness, and energy.

Parameter	*H* (*σ*)	*S* (*σ*)	*δ* (*σ*)	*ε_wl_* (*ε*)	*ρ_w_* (*ρ*)
Value	10 ~ 100	5 ~ 20	0.5 ~ 2.75	1 ~ 4	1 ~ 4
